# Renal vascular calcification and response to renal nerve denervation in resistant hypertension

**DOI:** 10.1097/MD.0000000000006611

**Published:** 2017-04-28

**Authors:** Annemiek F. Hoogerwaard, Mark R. de Jong, Ahmet Adiyaman, Jaap Jan J. Smit, Peter P.H.M. Delnoy, Jan-Evert Heeg, Boudewijn A.A.M. van Hasselt, Anand R. Ramdat Misier, Arif Elvan

**Affiliations:** Departments of Cardiology, Internal Medicine and Radiology, Isala Hospital, Zwolle, The Netherlands.

**Keywords:** arterial stiffness, renal sympathetic nerve denervation, treatment resistant hypertension, vascular calcification

## Abstract

Renal sympathetic nerve denervation (RDN) is accepted as a treatment option for patients with resistant hypertension. However, results on decline in ambulatory blood pressure (BP) measurement (ABPM) are conflicting. The high rate of nonresponders may be related to increased systemic vascular stiffness rather than sympathetic overdrive. A single center, prospective registry including 26 patients with treatment resistant hypertension who underwent RDN at the Isala Hospital in the Netherlands. Renal perivascular calcium scores were obtained from noncontrast computed tomography scans. Patients were divided into 3 groups based on their calcium scores (group I: low 0–50, group II: intermediate 50–1000, and group III: high >1000). The primary end point was change in 24-hour ABPM at 6 months follow-up post-RDN compared to baseline. Seven patients had low calcium scores (group I), 13 patients intermediate (group II), and 6 patients had high calcium scores (group III). The groups differed significantly at baseline in age and baseline diastolic 24-hour ABPM. At 6-month follow-up, no difference in 24-hour systolic ABPM response was observed between the 3 groups; a systolic ABPM decline of respectively −9 ± 12, −6 ± 12, −12 ± 10 mm Hg was found. Also the decline in diastolic ambulatory and office systolic and diastolic BP was not significantly different between the 3 groups at follow-up. Our preliminary data showed that the extent of renal perivascular calcification is not associated with the ABPM response to RDN in patients with resistant hypertension.

## 1 Introduction

Renal sympathetic nerve denervation (RDN) has been accepted as a treatment option for patients with resistant hypertension.^[1,2]^ However, the first, large, randomized, sham-controlled trial failed to demonstrate a benefit of RDN on reduction in 24-hour ambulatory blood pressure (BP) measurement (ABPM) at follow-up.^[3]^ Furthermore, even in the positive studies, 15% to 30% of treated patients were nonresponders to RDN.^[4–6]^ Therefore, it is thought that only a subgroup of patients will benefit from RDN. Studies have attempted to identify patient profiles likely to benefit from RDN. However, apart from baseline BP, no reliable predictor of response has yet been identified.^[7]^ Response to RDN in patients with resistant hypertension associated with stiff calcified arteries is not completely delineated. Studies have shown that isolated systolic hypertension is associated with increased calcium deposition in the aorta, and this is most marked in individuals who are resistant to antihypertensive therapy.^[8]^ Media calcification of the aorta leads to increased pulse wave velocity, elevated pulse pressure, and systolic hypertension.^[9–11]^ Furthermore, arterial stiffness is an independent predictor of total and cardiovascular morbidity and mortality.^[12]^ In this study, we aimed to explore whether the amount of aortic-renal vascular calcification, measured by the renal perivascular calcium score, affects the ABPM response to RDN. We hypothesized that nonresponse to RDN may be, at least in part, related to the extent of systemic vascular calcification rather than sympathetic overdrive.

## 2 Methods

### 2.1 Patient population, in- and exclusion criteria

We used our single center, prospective registry of 78 patients with treatment resistant hypertension who underwent RDN in the period of April 2012 till January 2015 at the Isala Hospital in the Netherlands. RDN was performed as an accepted treatment option for patients with resistant hypertension. Collection of data for this study was approved by the institutional board of the Isala Hospital. Computed tomography (CT) scans were performed as standard clinical work up before RDN. Patients in this registry were included and underwent RDN if they were aged between 18 and 80 years, had baseline systolic ABPM ≥ 140 mm Hg or diastolic ABPM ≥ 90 mm Hg despite stable antihypertensive treatment of at least 3 antihypertensive drugs (preferably including a diuretic) for at least 1 month. Patients were screened for eligibility for RDN by a multidisciplinary team, including: cardiologists, internists specialized in hypertension treatment, and a radiologist. Glomerular filtration rate had to be >45 mL/min/1.73 m^2^ according to the modification of diet in renal disease formula. Patients with secondary causes of hypertension, a history of renal artery stenosis or abnormal renal artery anatomy (assessed by CT—angiography), diabetes mellitus type 1, chronic oxygen use, or contraindication-to-anticoagulation therapy or heparin were excluded. Only in the last 26 patients in our registry, a noncontrast CT-scan was performed in order to determine the renal perivascular calcium scores for this study.

### 2.2 Renal perivascular calcium score

**Figure 1:**
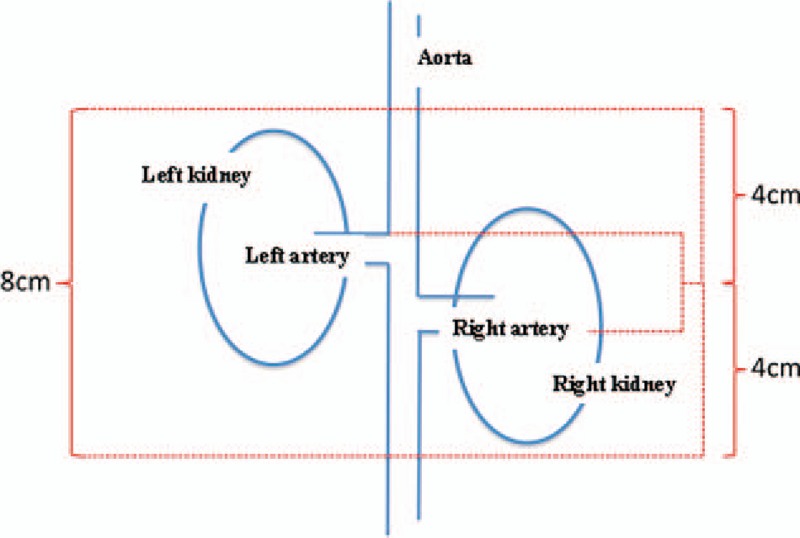
Renal perivascular calcium score assessment. cm = centimeters.

**Figure 2:**
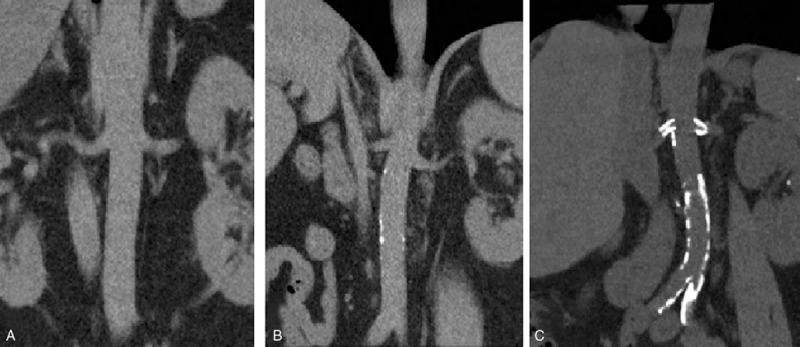
Computed tomography-angiography reconstruction of the renal arteries and part of the aorta of 3 patients with respectively a low (A), intermediate (B), or high calcium score (C). The ambulatory blood pressure measurements response for these patients 6 months post-RDN was respectively: −17 mmHg (A), −25 mmHg (B), and −13 mmHg (C).

The renal perivascular calcium score was used as a measurement to quantify the extent of vascular calcification. The calcium scores were obtained from noncontrast CT scans by dedicated radiology technicians of the radiology department at the Isala Hospital. These scans were performed before RDN before the CT angiography as part of the standard clinical work up to determine whether the patients’ renal artery anatomy was suitable for RDN. Quantitative calcium scores were calculated according to the method described by Agatson et al.^[13]^ The scores were determined using 8 cm of the perirenal abdominal aorta as depicted in Fig. 1. Eight centimeters were chosen, because the ostia of the left and right renal arteries had to be included in the part of the abdominal aorta. None of the screened patients had more than 8 cm in between the ostia of the 2 renal arteries. The patients were empirically divided into 3 groups based on their calcium scores: group I low calcium scores (0–50), group II intermediate calcium scores (50–1000), and group III high calcium scores (>1000). Figure 2 represents the CT-angiography reconstruction of the renal arteries and part of the aorta of 3 patients with respectively a low (I), intermediate (II), or high calcium score (III).

### 2.3 End points

The primary end point was decline in 24-hour systolic ABPM at 6-month follow-up post-RDN compared to baseline. Secondary end point was decline in diastolic ABPM, systolic and diastolic office BP at 6-month follow-up post-RDN.

### 2.4 RDN procedure and follow-up

To obtain access to the renal arteries, the right femoral artery was punctured, and via a Seldinger technique a guide wire was introduced. A pigtail catheter was introduced through the sheath, and a contrast angiography of the abdominal aorta was performed depicting the renal arteries. Subsequently, the renal artery was selectively cannulated with the renal ablation catheter (Symplicity, Medtronic, Minneapolis, MN or EnligHTN, St. Jude, Medical, St. Paul, MN), through a guiding sheath. The ablation catheter was introduced up to the first bifurcation and radiofrequency ablation lasting up to 2 minutes each and <8 W were applied in a spiral pattern, with 4 to 12 ablations within each renal artery, and 0.5-cm distance between ablation points. During the procedure heparin was given intravenously to obtain an activated clotting time 250 to 300 seconds. The patients were hospitalized for 1 night observation post intervention and were discharged the next day if no medical problems were observed. Follow-up after RDN at 6 months consisted of repeated blood tests, evaluation of antihypertensive medication, ABPM and office BP measurements performed at the outpatient clinic of the division of the vascular medicine at the Isala Hospital. Antihypertensive drug therapy was left unchanged, unless symptomatic hypotension or out of range hypertension (> 180 mm Hg systolic BP) warranting immediate control were present.

### 2.5 Statistical analysis

Statistical analysis was performed using IBM SPSS statistics version 20 (IBM inc., Armon, NY). Continuous variables were expressed as mean ± standard deviation or median with range when appropriate. Categorical variables were reported by frequencies and percentages. Response to RDN is defined as a decline of ≥5 mm Hg ABPM at 6-month follow-up. To compare the baseline characteristics and follow-up data of the 3 groups a Fisher exact test was used for the categorical variables and one-way between-groups analysis of variance (ANOVA) with post-hoc tests was used for the continuous variables. A *P* value of ≤0.05 was considered statistically significant.

## 3 Results

### 3.1 Baseline characteristics

**Figure 3:**
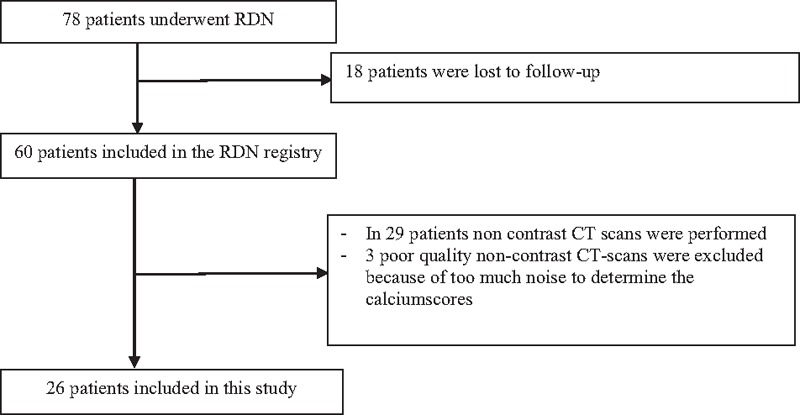
Flowchart. Inclusion of our study population.

Twenty-six patients with resistant hypertension were included in this study (Fig. 3). Seven patients had low calcium scores (0–50), 13 patients had intermediate calcium scores (50–1000), and 6 patients had high calcium scores (>1000). The baseline clinical characteristics, BP levels, and antihypertensive treatment of the 3 groups are shown in Table 1. A one-way between-groups ANOVA was conducted to explore the differences in continuous clinical variables. The groups differed statistically significant at baseline in age (group I: 54 ± 7, group II: 61 ± 9, group III: 69 ± 5, *P* = 0.005). Post-hoc comparisons using the Tukey honest significant difference test indicated that the mean age of group I was significantly different from group III. Group II did not differ significantly from either group I or III. The 3 groups did not differ significantly in proportion males, body-mass index, and medical history or for the number and type of medication used. The baseline diastolic 24-hour ABPM differed significantly in the 3 groups; respectively group I, II, and III: 103 ± 12, 89 ± 13, 86 ± 12 mm Hg (*P* = 0.03) as well as the baseline daytime diastolic ABPM (108 ± 11, 92 ± 14, 88 ± 11 mm Hg, *P* = 0.01). Again post-hoc comparison showed that group I differed significantly from group III for these baseline BP variables, and group II did not differ significantly from the other groups.

**Table 1:**
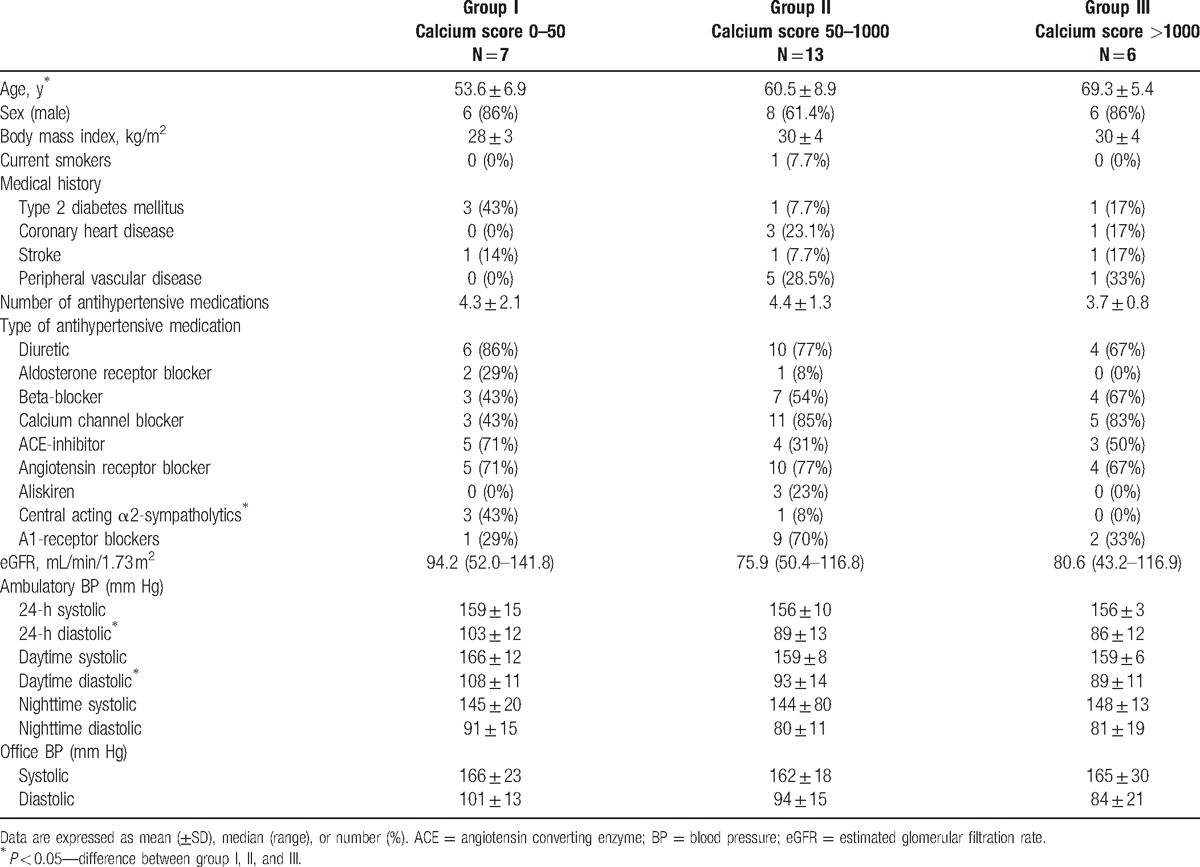
Baseline clinical characteristics of patients with low (group I), intermediate (group II), or high (group III) calcium scores.

### 3.2 Ambulatory blood pressure response at 6-month follow-up post-RDN

**Figure 4:**
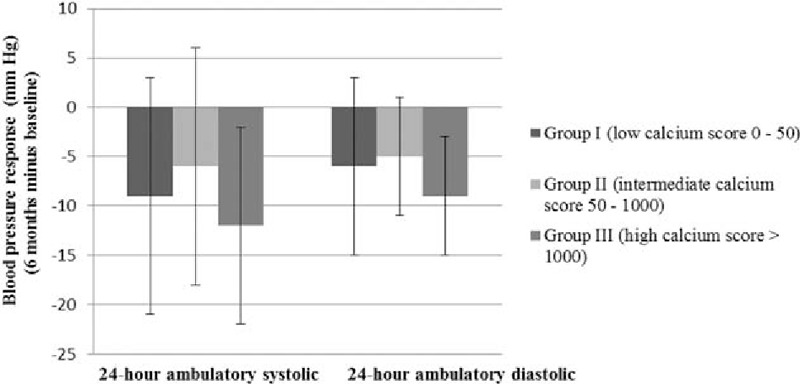
Ambulatory blood pressure response at 6 months of follow-up post-RDN in 3 patient groups based on their calcium scores (I: low, II: intermediate, and III: high).

In Fig. 4, the decline in ABP response at 6-month follow-up post-RDN is presented.

At 6-month follow-up 16 (61.5%) of the 26 patients were responders to RDN. Six-month post-RDN group I had a systolic ABP of 150 ± 10 mm Hg, group II 150 ± 9 mm Hg, and group III 144 ± 11 mm Hg. This represents a systolic ABP decline in the mentioned groups compared to baseline of respectively −9 ± 12 (*P* = 0.08), −6 ± 12 (*P* = 0.11), −12 ± 10 mm Hg (*P* = 0.03). A one-way between-groups ANOVA was conducted to compare the impact of the calcium scores on the decline in 24-hour systolic BP 6 months post-RDN. The 24-hour systolic ABP response was not statistically significant different between the 3 groups (*P* = 0.36) (Fig. 4). The decline in diastolic ABPM (respectively group I, II, and III: −6 ± 9, −5 ± 6, −9 ± 6 mm Hg, *P* = 0.61) (Fig. 4) was neither statistically significantly different between the 3 groups at 6-month follow-up.

### 3.3 Office blood pressure response at 6-month follow-up post-RDN

**Figure 5:**
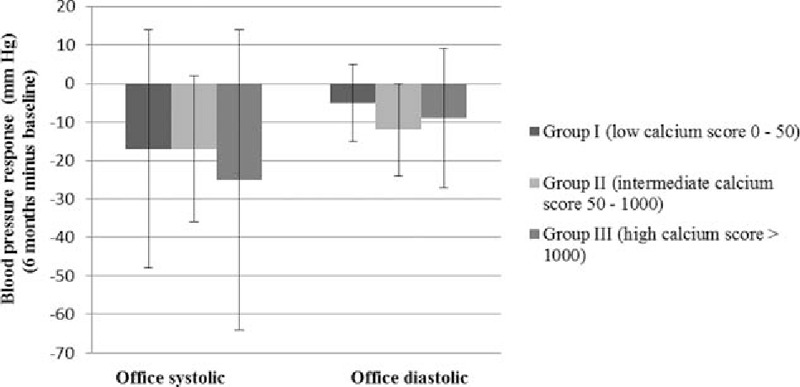
Office blood pressure response at 6 months of follow-up post-RDN in 3 patient groups based on their calcium scores (I: low, II: intermediate, and III: high).

The secondary end point was change in office BP measurements post-RDN. The decline in office systolic (group I: −17 ± 31, group II: −17 ± 19, group III: −25 ± 39 mm Hg, *P* = 0.78) as well as the decline in office diastolic BP (DBP) (group I: −5 ± 10, group II: −12 ± 12, group III −9 ± 18 mm Hg, *P* = 0.59) (Fig. 5) was not statistically significantly different between the 3 groups at 6-month follow-up.

## 4 Discussion

We report for the first time on the extent of vascular calcification and 24-hour ABPM response to RDN in patients with resistant hypertension. We hypothesized that patients with advanced vascular calcification will respond less to RDN due to vascular stiffness contributing more to maintaining hypertension rather than enhanced sympathetic tone. Our 3 groups of patients with various levels of vascular calcification differed significantly at baseline in age, that is, patients in the high calcium score group were significantly older compared to the low calcium score group. This in line with the literature describing aging as a major cause of vascular calcification.^[10]^ The baseline difference in DBP can be explained by increased vascular stiffness leading to lower DBP. In contrast to our hypothesis, based on the limited number of patients in this study, the extent of vascular calcification does not seem to be associated with either ambulatory or office BP response to RDN. Therefore, aiming to identify the patient's profile that will or will not benefit from RDN, we believe that patients with advanced vascular calcification do not have to be excluded from renal denervation therapy. Of note, a major limitation of this study is the small number of patients in all 3 groups and the very broad range in renal perivascular calcium scores (from 0 till above 1000) that we divided empirically into 3 groups. However, the wide range in calcium scores underlines our idea that different pathophysiological mechanisms are involved in maintaining hypertension and moreover in the ABPM response to RDN. In our study, at 6-month follow-up 16 (61.5%) of the 26 patients were responders to RDN. The systolic ABPM response 6-month post-RDN varied tremendously and ranged from + 8 to −22 mm Hg. We showed that some of these patients have clear calcified arteries (Fig. 2C), while others have no (Fig. 2A) or less (Fig. 2B) calcifications even though they have the same range of systolic hypertension before RDN (mean systolic ABPM at baseline group I: 166 ± 12, II: 159 ± 8, III: 159 ± 3 mmHg, *P* = 0.69). While other methods exist to score renal artery calcification, none have been compared head-to-head or have been validated in a larger cohort.^[14]^ Theoretically, arterial stiffness due to atherosclerosis might still be a predictor of nonresponse to RDN, but we did not find significant differences in our 3 defined groups of calcium score. We postulate that advanced vascular calcification may lead to incomplete RDN due to inadequate catheter electrodes to perivascular renal nerve contact.^[15,16]^ Or it could be the other way around those patients with low calcium scores will not respond to RDN because of inadequate catheter – renal nerve tissue contact due to for example noncalcified atherosclerotic plaques or large distance from endovascular wall to the nerve tissue in the adventitia which might lead to inadequate ablations.^[17]^ Vink et al^[18]^ have shown that RDN does not always result in circular lesions completely destroying the renal nerves. These considerations highlight, apart from exploring predictors of response, the need for a clear procedural end point of RDN. Chinushi et al^[19]^ showed in 8 dogs that electrical autonomic nerve stimulation of the renal artery leads to increased systemic BP, and simultaneously to changes in serum catecholamine and heart rate variability suggesting that the induced increase in BP was due to sympathetic nervous activity. On top of that, these effects to electrical stimulations were blunted after RDN. Gal et al^[20]^ confirmed these observations in patients undergoing RDN for resistant hypertension and demonstrated that high-frequency electrical renal nerve stimulation is feasible and results in acute, temporary increase in BP, which was blunted after RDN.

In conclusion, based on our preliminary data, the extent of vascular calcification is not associated with the ABPM response to RDN, and retrospectively apart from baseline 24-hour systolic ABPM, no other predictor of response was found in our study population. Future research should be directed in identifying predictors of response to RDN and creating a procedural end point for RDN. Furthermore, the role of vascular calcification in patients with resistant hypertension undergoing RDN needs to be investigated in a larger group of patients.
